# Antibacterial Activity of Berberine Against *Aeromonas hydrophila* and Associated Transcriptional Reprogramming

**DOI:** 10.3390/biology15141177

**Published:** 2026-07-17

**Authors:** Lianshou Lu, Jian Zhang, Xianming Liang, Dongkai Wang

**Affiliations:** 1 China Institute of Veterinary Drug Control, No. 8 South Zhongguancun Street, Haidian District, Beijing 100081, China; lutianxin2000@sina.com; 2 Department of Pharmaceutics, School of Pharmacy, Shenyang Pharmaceutical University, 103 Wenhua Road, Shenyang 110016, China; zhangjworks@163.com

**Keywords:** berberine, *Aeromonas hydrophila*, antibacterial mechanism, transcriptomics, energy metabolism

## Abstract

Berberine (BBR), a plant-derived isoquinoline alkaloid, exhibits broad-spectrum antibacterial activity, but its molecular mechanisms against *Aeromonas hydrophila* remain unclear. This study investigated the antibacterial action and underlying mechanisms of BBR against *A. hydrophila* through integrated phenotypic assays and transcriptomic analysis. BBR showed notable antibacterial activity with a minimum inhibitory concentration (MIC) of 2.5 g/L. Treatment at a sub-inhibitory concentration (1/2 MIC) severely compromised cell membrane integrity, evidenced by increased leakage of alkaline phosphatase (AKP) and β-galactosidase (β-GAL). Transmission electron microscopy (TEM) revealed ultrastructural damage including plasmolysis and membrane rupture. RNA-seq analysis identified 740 differentially expressed genes (DEGs). Crucially, BBR extensively downregulated core energy metabolism and catabolic pathways, including the TCA cycle, fatty acid β-oxidation, and amino acid degradation. Concurrently, genes associated with flagellar assembly and DNA repair were upregulated. These findings reveal that BBR exerts its antibacterial effect via a multi-target mechanism involving direct physical damage to the cell envelope and the suppression of central metabolism. This study elucidates the antibacterial effects of BBR against *A. hydrophila* and provides a foundation for its potential use as an eco-friendly agent in aquaculture.

## Simple Summary

Bacterial diseases pose a significant challenge to sustainable aquaculture production worldwide and cause substantial economic losses for fish farmers. *Aeromonas hydrophila* is a widespread harmful bacterium that infects farmed fish and spreads readily in aquaculture environments. The long-term overuse of conventional antibiotics has led to growing bacterial drug resistance, creating an urgent need for safe and effective natural alternatives. This study assessed the antibacterial effect of berberine, a natural substance from medicinal plants, against this bacterium, and explored how it influences the bacterium’s internal gene activity. We found that berberine effectively inhibits the growth of *Aeromonas hydrophila*. It works by altering the activity of numerous bacterial genes, which disrupts the bacterium’s energy production, cell structure formation, and disease-causing ability. These findings indicate that berberine is a promising natural antibacterial agent for protecting farmed fish, and can support the development of more eco-friendly and sustainable aquaculture.

## 1. Introduction

*Aeromonas hydrophila* is a zoonotic pathogen widely distributed in aquatic environments, capable of causing septicemia, skin ulcers, and intestinal infections in aquatic animals [1]. These diseases lead to severe economic losses in the aquaculture of commercially important species such as grass carp (*Ctenopharyngodon idella*), crucian carp (*Carassius auratus*), largemouth bass (*Micropterus salmoides*), salmon, and crabs [2,3,4]. For decades, antibiotics including enrofloxacin and florfenicol have been widely used to prevent and treat bacterial diseases in aquaculture [5]. However, their overuse and misuse have not only fostered the emergence of drug-resistant bacterial strains but also raised significant environmental and food safety concerns due to residue persistence [6]. This dual crisis highlights the critical demand for novel, eco-friendly antibacterial agents.

Berberine (BBR) is a naturally occurring isoquinoline alkaloid extracted from medicinal plant species including *Phellodendron amurense* and *Coptis chinensis* [7]. It has garnered significant attention as a promising alternative to conventional antibiotics, owing to its broad-spectrum anti-inflammatory, antioxidant, and antimicrobial activities [8]. Studies have demonstrated its efficacy against various economically significant aquatic pathogens, including *Edwardsiella ictaluri*, *Streptococcus agalactiae*, and *A. hydrophila* [9,10,11]. Research has begun to elucidate its antibacterial mechanisms, indicating that BBR may damage the cell membrane of *A. hydrophila*, increasing permeability and altering membrane fluidity [12]. Beyond its direct antimicrobial action, dietary BBR supplementation confers significant health benefits to aquatic hosts. For instance, in *Charybdis japonica* (Asian paddle crab) infected with *A. hydrophila*, BBR treatment enhanced survival, modulated gut microbiota, and improved intestinal barrier function [13,14]. Similar improvements in glucose metabolism, antioxidant capacity, and gut health have been observed in *Nile tilapia* and largemouth bass fed BBR-supplemented diets [15,16,17]. Furthermore, BBR can act as an antibiotic adjuvant, potentially reversing multidrug resistance by inhibiting bacterial efflux pumps [18]. Despite these advancements, current research has predominantly focused on the immunomodulatory effects of BBR on the host, while a comprehensive understanding of its direct antibacterial mechanisms against *A. hydrophila* at the molecular level remains limited.

Transcriptomic approaches have proven instrumental in deciphering the global molecular responses of bacteria to antimicrobial agents, revealing complex stress adaptations and metabolic reprogramming. Multi-omics studies have elucidated BBR’s effects on bacteria such as *Escherichia coli* and *Staphylococcus aureus* [12,19]. These effects include the suppression of key metabolic pathways and disruption of cell wall integrity. In contrast, studies on *A. hydrophila* have primarily focused on phenotypic antibacterial activity or host immune responses [9,10,11], while the direct transcriptional response of the pathogen to BBR remains largely uncharacterized. To bridge this knowledge gap, the present study reveals for the first time the genome-wide transcriptional reprogramming of *A. hydrophila* under BBR treatment and systematically elucidates the multi-target antibacterial mechanism, from cell envelope damage to central metabolic suppression. Elucidating this response is critical to understanding how BBR reprograms bacterial virulence, survival strategies, and core physiological functions.

## 2. Materials and Methods

### 2.1. Chemicals and Bacterial Strain

Berberine (BBR) of high-performance liquid chromatography (HPLC) grade (≥98% purity) was procured from Shanghai Yuanye Bio-Technology Co., Ltd. (Shanghai, China). Resazurin sodium salt, o-nitrophenyl-β-D-galactopyranoside (ONPG), and all other analytical-grade chemical reagents were acquired from Sigma-Aldrich (St. Louis, MO, USA). The *A. hydrophila* strain S3 was routinely propagated in Tryptic Soy Broth (TSB; BD Difco^TM^ (Sparks, MD, USA)) under continuous orbital shaking at 180 rpm and 28 °C. Tryptic Soy Agar (TSA) was utilized for solid-phase cultivation. Bacterial cultures in the mid-logarithmic growth phase, corresponding to an optical density at 600 nm (OD_600_) of approximately 0.6, were employed for all downstream experiments.

### 2.2. Determination of Minimum Inhibitory and Bactericidal Concentrations

The antibacterial potency of BBR against *A. hydrophila* was quantified via its minimum inhibitory concentration (MIC) and minimum bactericidal concentration (MBC). A modified resazurin-based broth microdilution method, using resazurin as a bacterial metabolic viability indicator, was adopted for the MIC assay with reference to a published protocol [20]. In brief, TSB medium was used to adjust the bacterial suspension to a final density of 1 × 10^5^ CFU/mL. Serial two-fold dilutions of BBR, covering a concentration range from 0.16 to 20 g/L, were dispensed into a 96-well microplate, followed by the addition of the standardized bacterial inoculum to each well. Wells filled with TSB medium supplemented with 1% DMSO (the solvent for BBR stock solution) without bacteria were set as the blank negative control, while wells containing only bacterial suspension in TSB served as the growth positive control. Following a 24 h incubation at 28 °C, 50 µL of 0.01% (*w*/*v*) resazurin working solution was added to each well. The plates were incubated for an additional 4 h under the same temperature condition. The MIC value was defined as the lowest BBR concentration at which no color transition from blue to pink was observed. For MBC quantification, 100 µL aliquots were withdrawn from wells with no visible color change (i.e., concentrations at or above the MIC) and spread evenly on TSA agar plates. After 24 h of incubation at 28 °C, the MBC was identified as the lowest BBR concentration that yielded no viable bacterial colonies on the plates. All assays were carried out with three independent biological replicates.

### 2.3. Assessment of Cell Membrane Permeability

The disruptive effect of BBR on *A. hydrophila* cell envelope integrity was evaluated by quantifying the leakage of intracellular alkaline phosphatase (AKP) and β-galactosidase (β-GAL), enzymes typically located in the periplasmic space and cytoplasm, respectively. Bacterial cultures were exposed to BBR at 1/2 MIC (1.3 g/L) for 6 h at 28 °C with shaking, with untreated cells set as the control. After incubation, samples were centrifuged at 8000× *g* for 10 min at 4 °C, and supernatants were collected for enzyme activity detection. Extracellular AKP activity was determined using a commercial assay kit following the manufacturer’s protocol, with results expressed in U/L. For β-GAL quantification, ONPG was used as the reaction substrate: 100 µL of supernatant was mixed with 900 µL of Z-buffer (pH 7.0) containing 0.6 mg/mL ONPG, incubated at 37 °C for 30 min, and terminated by adding 500 µL of 1 M Na_2_CO_3_. β-GAL activity was calculated by measuring the absorbance of the released o-nitrophenol (ONP) at 420 nm. All assays were conducted with three independent biological replicates.

### 2.4. Transmission Electron Microscopy Analysis

Ultrastructural morphological damage induced by BBR in *A. hydrophila* cells was visualized via transmission electron microscopy (TEM). Bacterial cultures were treated with BBR at 1/2 MIC (1.3 g/L) for 6 h, and untreated cultures served as the control. Cell pellets harvested by centrifugation (8000× *g*, 10 min) were washed twice with sterile 0.1 M phosphate-buffered saline (PBS, pH 7.4), then pre-fixed with 2.5% (*v*/*v*) glutaraldehyde at 4 °C overnight. Following three washes with PBS, the samples were post-fixed with 1% (*w*/*v*) osmium tetroxide for 2 h at room temperature. Subsequently, the samples were dehydrated through a graded ethanol series (30%, 50%, 70%, 80%, 90%, 95%, and 100%), infiltrated, and embedded in epoxy resin (SPI-PON 812). Ultrathin sections were prepared with a Leica UC7 ultramicrotome (Leica Microsystems, Wetzlar, Germany), double-stained with uranyl acetate and lead citrate, and examined under a Hitachi HT7800 TEM system (Hitachi High-Tech Corporation, Hitachinaka, Japan) operated at an accelerating voltage of 80 kV.

### 2.5. Biofilm Formation Assay

The effect of BBR on biofilm formation was assessed using the crystal violet staining method. Overnight culture of *A. hydrophila* was diluted to 1 × 10^6^ CFU/mL in fresh TSB medium. The bacterial suspension was mixed with BBR to a final concentration of 1/2 MIC, while an equal volume of sterile water was added to the untreated control group (CK). Aliquots of 200 μL were added to 96-well polystyrene plates and incubated statically at 28 °C for 24 h. Planktonic cells were gently removed, and wells were washed three times with sterile PBS. Adherent biofilms were fixed with methanol for 15 min, stained with 0.1% (*w*/*v*) crystal violet for 10 min, and destained with 33% (*v*/*v*) glacial acetic acid. Absorbance was measured at 570 nm. Three biological replicates were performed per group.

### 2.6. Transcriptome Analysis

#### 2.6.1. Sample Preparation, RNA Extraction and Sequencing

To capture the global transcriptional response, *A. hydrophila* cultures (1 × 10^7^ CFU/mL) were treated with BBR at 1/2 MIC for 6 h. An untreated culture served as the control (CK). This condition was selected to induce substantial transcriptional reprogramming while maintaining high cell viability, as cells at 6 h were in mid-logarithmic phase with active and stable physiological status under our culture conditions. This exposure time and concentration were chosen to induce a substantial transcriptional shift while maintaining sufficient cell viability for RNA extraction. For each group, five independent biological replicates were prepared. Total RNA was extracted using TRIzol^®^ Reagent (Invitrogen, Carlsbad, CA, USA) following the manufacturer’s standard protocol. RNA concentration and purity were determined spectrophotometrically with a NanoDrop 2000 instrument (Thermo Fisher Scientific, Waltham, MA, USA). Ribosomal RNA was depleted using the Ribo-off rRNA Depletion Kit (Vazyme, Nanjing, China), after which sequencing libraries were constructed with the NEBNext^®^ Ultra^TM^ II RNA Library Prep Kit (NEB, Ipswich, MA, USA). Paired-end sequencing with a read length of 150 bp was carried out on the Illumina NovaSeq X Plus platform (Illumina, San Diego, CA, USA).

#### 2.6.2. Bioinformatic Analysis of RNA-seq Data

Prior to downstream analysis, raw sequencing reads underwent quality control processing using fastp (v0.23.2) [21], in which adapter sequences were trimmed and bases with a quality score lower than Q20 were filtered out to yield high-quality clean reads. The filtered reads were then mapped against the reference genome of *A. hydrophila* strain ATCC 7966 (NCBI Assembly accession: GCF_000014805.1) with the Bowtie2 alignment tool (v2.4.5) [22]. For transcript abundance quantification, the RSEM package (v1.3.3) was applied to calculate gene expression levels, which were normalized to FPKM values. To evaluate the overall transcriptomic profile distribution and the reproducibility of biological replicates, principal component analysis (PCA) and unsupervised hierarchical clustering were performed based on genome-wide expression data. Differential expression analysis between the BBR treatment and CK groups was conducted using the DESeq2 R package (v1.38.3) [23]. Genes satisfying both thresholds of an FDR-corrected *p*-value < 0.05 and |log_2_ fold change| > 1 were designated as differentially expressed genes (DEGs). To elucidate the functional characteristics of these DEGs, Gene Ontology (GO) term and Kyoto Encyclopedia of Genes and Genomes (KEGG) pathway enrichment analyses were performed using the clusterProfiler R package (v4.6.2), with an adjusted *p*-value below 0.05 defined as the significance cutoff.

### 2.7. Quantitative Real-Time PCR (RT-qPCR) Validation

To verify the reliability of the RNA-seq expression profiles, ten differentially expressed genes representing distinct functional categories (*ndk*, *flgE*, *rtxB*, *recA*, *fliC*, *hisC*, *katE*, *aerA*, *sucA*, *ompF*) were selected at random for RT-qPCR validation. Primer pairs targeting these genes were designed using the NCBI Primer-BLAST tool (https://www.ncbi.nlm.nih.gov/tools/primer-blast/, accessed on 19 March 2026), with full sequences provided in Table 1. First-strand cDNA was synthesized from 1 μg total RNA with HiScript III RT SuperMix (+gDNA wiper, Vazyme, Nanjing, China). qPCR was conducted on a QuantStudio 5 system (Applied Biosystems, Austin, TX, USA) using ChamQ SYBR Master Mix (Vazyme, Nanjing, China). Each 20 μL reaction included 10 μL 2× Master Mix, 0.4 μL each primer (10 μM), 2 μL 10-fold diluted cDNA, and 7.2 μL nuclease-free water. The cycling protocol comprised initial denaturation at 95 °C for 30 s and 40 subsequent cycles of 95 °C for 10 s plus 60 °C for 30 s. The 16S rRNA gene served as the internal reference, and relative expression was calculated via the 2^−ΔΔCt^ method with three biological and technical replicates.

### 2.8. Statistical Analysis

Data are reported as mean ± standard deviation. Prior to formal statistical analysis, the normality of the data distribution was assessed using the Shapiro–Wilk test, and homogeneity of variance was evaluated using Levene’s test. For comparisons between two groups with normal distribution and equal variance, an unpaired Student’s *t*-test was applied. For multiple group comparisons, one-way analysis of variance (ANOVA) was performed, followed by Tukey’s post hoc test for pairwise comparisons. *p* < 0.05 was set as the threshold for statistical significance.

## 3. Results

### 3.1. Determination of the MIC and MBC of BBR

**Figure 1:**
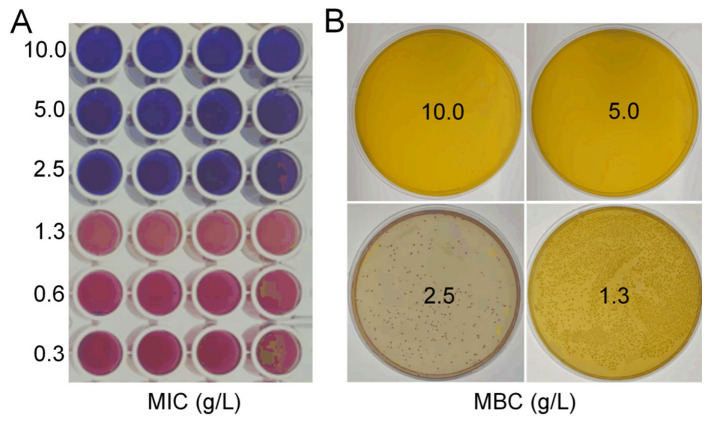
In vitro inhibitory effects of BBR against *A. hydrophila*. (**A**) MIC determination via the resazurin microdilution method. The well containing 2.5 g/L BBR shows no color change from blue to pink, defining the MIC. (**B**) MBC determination. No bacterial colonies were observed on the agar plate inoculated from the well containing 5.0 g/L BBR.

The in vitro antibacterial potency of berberine (BBR) against *A. hydrophila* was quantitatively assessed. Utilizing the resazurin-based broth microdilution assay, the MIC of BBR was determined to be 2.5 g/L (Figure 1A). To evaluate its bactericidal effect, aliquots from wells showing no bacterial growth were subcultured on agar plates. The MBC was identified as 5.0 g/L, as no viable colonies were recovered at this or higher concentrations (Figure 1B).

### 3.2. BBR Compromises the Membrane Integrity of A. hydrophila

**Figure 2:**
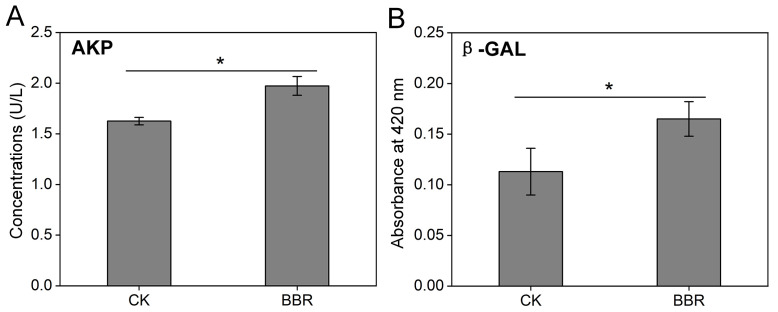
BBR-induced disruption of *A. hydrophila* membrane permeability. Extracellular activities of (**A**) alkaline phosphatase (AKP) and (**B**) β-galactosidase (β-GAL) were measured after treatment with BBR at the 1/2 MIC (1.3 g/L). AKP activity is expressed in U/L. β-GAL activity is represented by the absorbance at 420 nm (A_420_) resulting from the hydrolysis of the substrate ONPG. Values are presented as mean ± SD (*n* = 3). Significant differences versus the control group are marked with asterisks (*) at the *p* < 0.05 level.

To investigate whether BBR targets the bacterial cell envelope, its effect on membrane permeability was evaluated by measuring the leakage of intracellular enzymes. Following exposure to BBR at its 1/2 MIC (1.3 g/L), the activities of AKP and β-GAL were significantly elevated compared to those in the untreated control (CK) group (*p* < 0.05). AKP activity increased from 1.63 ± 0.04 U/L (CK) to 1.97 ± 0.09 U/L (BBR), while the absorbance at 420 nm resulting from the β-GAL-catalyzed ONPG hydrolysis increased from 0.11 ± 0.02 to 0.17 ± 0.02 (Figure 2). This marked increase in extracellular enzyme activities indicates that BBR treatment disrupts the integrity of the cell membrane, leading to the leakage of cytoplasmic contents.

### 3.3. BBR Induces Severe Ultrastructural Damage in A. hydrophila Cells

**Figure 3:**
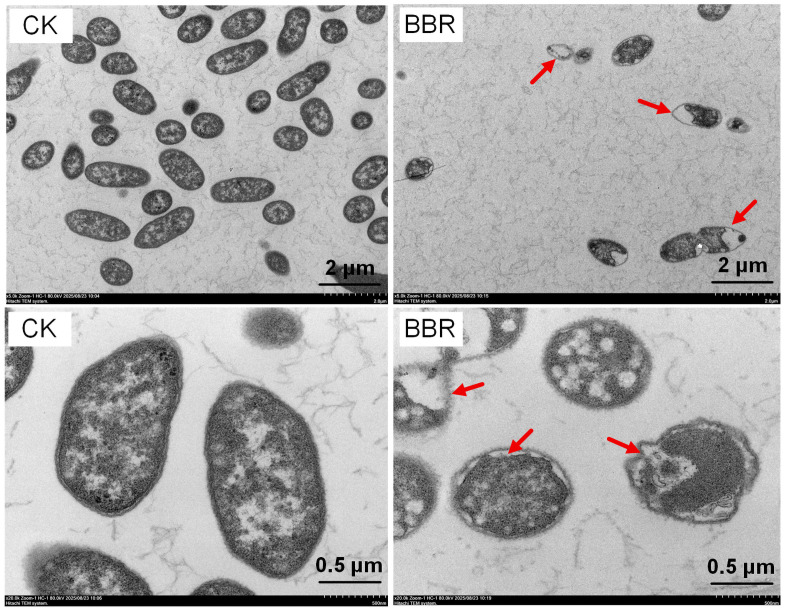
Ultrastructural alterations in *A. hydrophila* visualized by TEM. Control (CK) cells showing intact morphology. BBR-treated cells exhibiting severe damage (red arrows), including membrane rupture, plasmolysis, vacuolization, and cytoplasmic leakage.

TEM was employed to visualize the direct impact of BBR on bacterial cell morphology at the subcellular level. Untreated control cells (CK) exhibited a typical, intact Gram-negative structure, characterized by a smooth, continuous outer membrane, a well-defined periplasmic space, and uniformly dense cytoplasmic contents. In contrast, TEM analysis revealed that BBR-treated cells exhibited severe ultrastructural damage, characterized by evident plasmolysis (detachment of the membrane from the cell wall), local membrane rupture, intracellular vacuolization, and leakage of cytoplasmic contents resulting in uneven electron density (Figure 3). These observations provide direct visual evidence that BBR severely damages the cellular integrity of *A. hydrophila*.

### 3.4. Inhibitory Effect of BBR on Biofilm Formation of A. hydrophila

**Figure 4:**
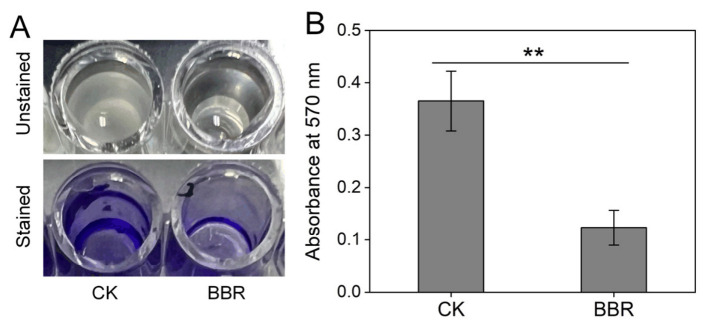
Inhibitory effect of BBR on biofilm formation of *A. hydrophila*. (**A**) Representative crystal violet-stained image of biofilms in a 96-well plate. (**B**) Quantification of biofilm formation by measuring absorbance at 570 nm. Values are presented as mean ± SD (*n* = 3). ** denotes statistically significant differences between the BBR and CK groups at *p* < 0.01.

Crystal violet staining showed that BBR significantly inhibited biofilm formation compared with the untreated control (Figure 4A). Quantitative analysis revealed that the OD_570_ value in the BBR-treated group was 0.12 ± 0.03, significantly lower than that in the control group (0.37 ± 0.06) (*p* < 0.05) (Figure 4B). This result indicates that BBR at a sub-inhibitory concentration can effectively suppress the biofilm-forming capacity of *A. hydrophila*.

### 3.5. Global Transcriptomic Response of A. hydrophila to BBR Treatment

#### 3.5.1. Overview of RNA-Seq Data

**Figure 5:**
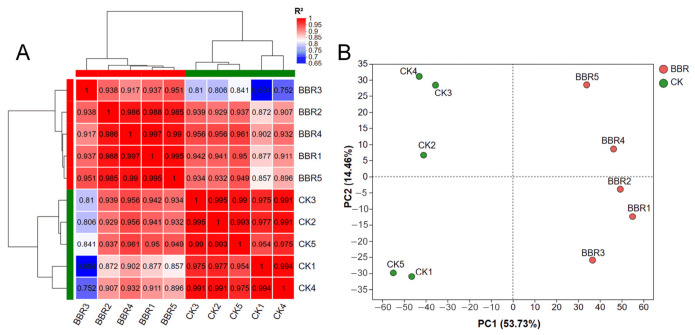
Global transcriptomic profile of *A. hydrophila* in response to BBR treatment. (**A**) Hierarchical clustering analysis of all expressed genes. (**B**) PCA showing distinct separation between BBR-treated and control (CK) samples.

**Figure 6:**
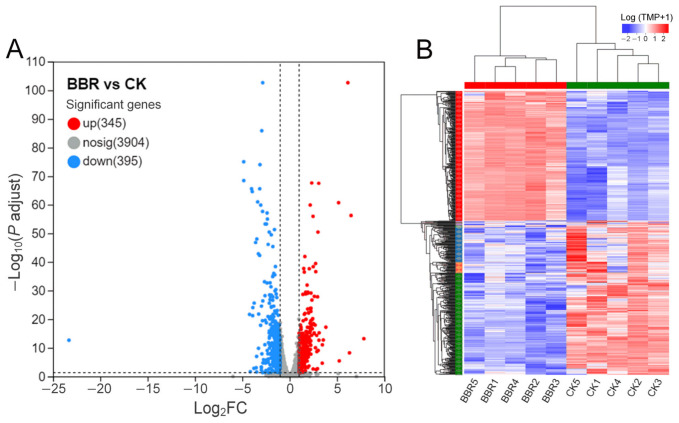
Overview of DEGs. (**A**) Volcano plot illustrating DEGs between the BBR and CK groups. Significantly upregulated and downregulated genes are marked in red and blue, respectively (|log_2_ fold change| > 1, FDR-corrected *p* < 0.05), while grey dots represent genes with no significant expression alteration. (**B**) Hierarchical clustering heatmap of all 740 DEGs. The color bar Log(TMP+1) displays normalized gene expression, with blue indicating low transcript abundance and red indicating high transcript abundance; samples are clearly clustered into the BBR-treated group and CK control group.

To elucidate the molecular mechanisms underlying BBR’s antibacterial action, a comparative transcriptomic analysis was conducted on *A. hydrophila* treated with BBR at 1/2 MIC versus an untreated control. After stringent quality control, each library yielded over 22.3 million clean reads. The high-quality metrics, including low base error rates (<0.012%), high Q20 scores (>99.2%), and high genome mapping rates (>92.9%), confirmed the reliability of the sequencing data for downstream analysis (Table 2). Hierarchical clustering revealed a clear separation between the two experimental groups (Figure 5A). Principal component analysis (PCA) further demonstrated a distinct segregation of BBR-treated samples from the control group along the first principal component (PC1), which accounted for 53.73% of the total variance (Figure 5B). These results indicate that BBR treatment induced a substantial global shift in gene expression. Differential expression analysis identified a total of 740 significantly dysregulated genes (*p* < 0.05, |log_2_FoldChange| > 1), comprising 345 upregulated and 395 downregulated genes in the BBR group (Figure 6A). Clustering analysis of these differentially expressed genes (DEGs) further reinforced the distinct transcriptional profiles between the two conditions (Figure 6B).

#### 3.5.2. GO Enrichment Analysis of DEGs

**Figure 7:**
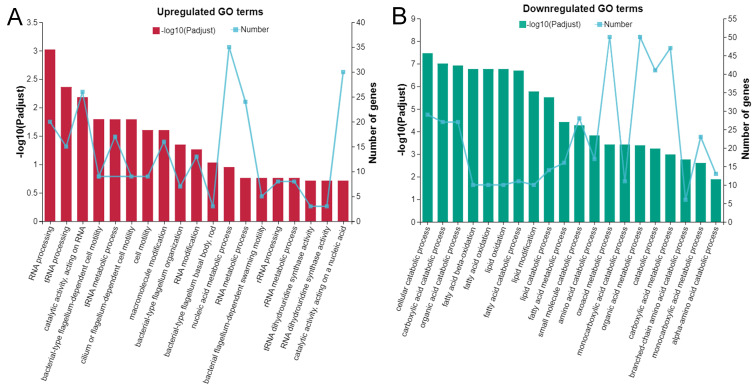
GO enrichment analysis of DEGs. The top 20 significantly enriched GO terms for (**A**) upregulated and (**B**) downregulated genes in the BBR group compared to the CK group.

GO enrichment analysis was performed to categorize the biological functions of the DEGs. For upregulated genes, the most significantly enriched terms were predominantly associated with RNA metabolic processes (e.g., RNA processing, tRNA metabolic process, rRNA processing) and cell motility (e.g., bacterial-type flagellum-dependent cell motility, swarming motility) (Figure 7A). In contrast, the downregulated gene set was predominantly enriched in catabolic and metabolic pathways, with a prominent focus on fatty acid oxidation (including fatty acid β-oxidation and lipid oxidation) and amino acid breakdown (including branched-chain and α-amino acid catabolic processes) (Figure 7B).

#### 3.5.3. KEGG Enrichment Analysis of DEGs

**Figure 8:**
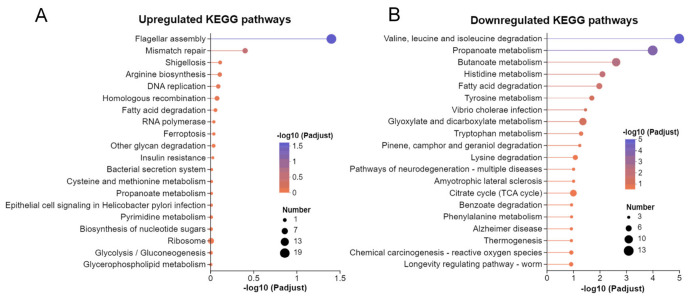
KEGG pathway enrichment analysis of DEGs. Lollipop plots displaying the top 20 significantly enriched pathways for (**A**) upregulated and (**B**) downregulated genes in the BBR-treated group. The dot size represents the number of genes, and the color scale corresponds to the -lg (adjusted *p*-value).

KEGG pathway analysis further delineated the systemic metabolic perturbations. Upregulated pathways in BBR-treated cells included Flagellar assembly, DNA replication and repair (Mismatch repair, Homologous recombination), and several biosynthesis pathways such as Arginine biosynthesis and Cysteine and methionine metabolism (Figure 8A). Strikingly, the most significantly downregulated pathways were centralized on energy metabolism and nutrient degradation. These suppressed pathways encompassed valine, leucine and isoleucine degradation, fatty acid catabolism, the tricarboxylic acid (TCA) cycle, and several additional amino acid degradation pathways (e.g., Histidine, Tryptophan, Lysine degradation) (Figure 8B). This coordinated suppression of core catabolic pathways suggests a severe disruption in the bacterium’s ability to generate energy and metabolic precursors.

### 3.6. Validation of RNA-Seq Data by RT-qPCR

**Figure 9:**
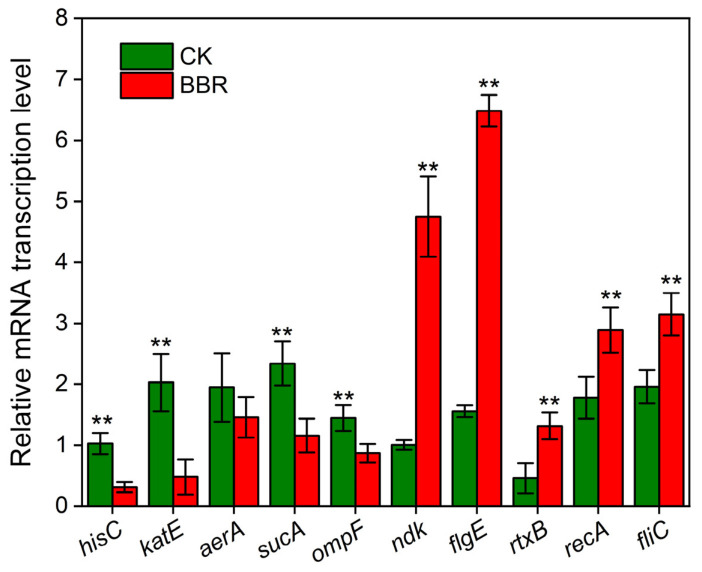
RT-qPCR validation of DEGs. Values are presented as mean ± SD (*n* = 3). ** denote statistically significant differences between the BBR and CK groups at *p* < 0.01.

To confirm the reliability of the transcriptome sequencing results, ten DEGs were selected for validation using RT-qPCR. The selected genes included five upregulated (*ndk*, *flgE*, *rtxB*, *recA*, *fliC*) and five downregulated (*hisC*, *katE*, *aerA*, *sucA*, *ompF*) targets. The RT-qPCR results exhibited a strong concordance with the RNA-seq data. Nine of the selected genes showed consistent expression patterns across the two detection approaches, with statistically significant expression differences between the BBR and CK groups (*p* < 0.05) (Figure 9). This high correlation validates the accuracy of the transcriptomic profiling.

## 4. Discussion

The plant-derived antimicrobial compound BBR represents a promising candidate as an antibiotic alternative in aquaculture due to its multi-target action and potential to mitigate resistance development [24]. BBR has demonstrated a broad spectrum of antibacterial activity against various pathogens through diverse mechanisms. For example, Gu et al. (2025) found that BBR could suppress wall teichoic acid biosynthesis and compromise cell wall integrity in methicillin-resistant *Staphylococcus aureus* [25]. Similarly, Du et al. (2020) demonstrated that BBR exerted its antibacterial effects against *Streptococcus pyogenes* by perturbing carbohydrate metabolism and inducing the accumulation of reactive oxygen species [26]. This study confirms its inhibitory effect against the aquatic pathogen *A. hydrophila*, with an MIC of 2.5 g/L and an MBC of 5.0 g/L, findings that are consistent with its known antibacterial role against other bacteria. In comparison with published data, the MIC value against *A. hydrophila* is at a similar level to that against other Gram-negative pathogens; for example, the reported MIC of berberine against *Pseudomonas aeruginosa* is approximately 2.0 g/L [27]. Beyond direct antibacterial activity, BBR has been observed to exhibit synergistic effects with conventional antibiotics such as quinolones, β-lactams, aminoglycosides, tetracyclines and macrolides, potentially reducing their required usage [18,28]. In addition, dietary supplementation with BBR may offer immunomodulatory and health-promoting benefits that extend beyond bactericidal action. For instance, studies in the Asian paddle crab (*Charybdis japonica*) have shown that BBR treatment can elevate the activities of immune and antioxidant enzymes, and improve intestinal barrier function [14,29]. These combined antibacterial, immunomodulatory, and health-promoting effects underscore BBR’s broad application potential as an eco-friendly agent in aquaculture.

The antibacterial mechanism of BBR is strongly linked to its disruptive effect on bacterial cell envelope integrity. In this study, treatment with BBR resulted in a significant increase in the extracellular activity of AKP and β-GAL, and TEM observation directly revealed ultrastructural damage including plasmolysis, membrane rupture, and cytoplasmic leakage. These phenotypic results provide direct experimental evidence for the membrane-damaging effect of BBR on *A. hydrophila*. This mechanism is well-supported by prior research on berberine against both *A. hydrophila* and other bacterial pathogens [12,27,30]. Notably, while earlier work mainly documented this membrane damage at the phenotypic level, our transcriptomic data further reveal concurrent dysregulation of genes involved in cell membrane biogenesis and cell wall assembly, providing molecular-level evidence to support this mechanism. Research focusing on *Pseudomonas aeruginosa* has revealed that berberine hydrochloride exerts its antimicrobial activity by disrupting bacterial cell membranes, inducing reactive oxygen species accumulation, and reducing intracellular ATP levels [27]. This mode of action, targeting the cell envelope, is not unique to BBR but represents a common and effective strategy employed by various antimicrobial compounds. For example, plant polyphenols have been reported to damage the cell membrane and wall integrity of *Vibrio alginolyticus*, resulting in increased AKP activity and protein leakage [31]. Similarly, *Macleaya cordata* alkaloids (sanguinarine and chelerythrine) have been shown to inhibit *Nocardia seriolae* by disrupting cell envelope integrity and increasing membrane permeability [30]. The consistency of this phenotype across different pathogens and antimicrobial compounds suggests that targeting the cell envelope is a common and effective strategy. Our results confirm that this mechanism is a central component of BBR’s antibacterial action against *A. hydrophila*.

Transcriptomic analysis provides a crucial systems-level perspective to decipher the molecular underpinnings of BBR’s antibacterial action against *A. hydrophila*. Our principal component analysis confirmed a profound and global reprogramming of the bacterial transcriptome upon BBR exposure. This large-scale transcriptional shift aligns with multi-omics studies on BBR against other pathogens, such as *Escherichia coli*, where BBR was found to interfere with a wide array of cellular processes [12]. The most dominant pattern emerging from our data is the coordinated and extensive downregulation of central energy metabolism and catabolic pathways. Genes encoding key enzymes in the TCA cycle, fatty acid β-oxidation, and the degradation of multiple amino acids (e.g., valine, leucine, isoleucine) were significantly suppressed. These observed transcriptional changes suggest that BBR treatment may impair bacterial energy production capacity and potentially induce a bioenergetic deficit, which would deplete intracellular ATP pools and deprive the cell of essential biosynthetic precursors required for growth, replication, and cellular integrity maintenance [32]. Notably, this inference is drawn from transcriptomic pathway enrichment analysis and has not been directly validated by physiological assays. This mechanism of “metabolic starvation” is a recognized antibacterial tactic and is consistent with proteomic findings in *Streptococcus pyogenes*, where BBR treatment perturbed carbohydrate and fatty acid metabolism [26]. Furthermore, a cell metabolomics study suggested that BBR can target citrate metabolism, affecting downstream catabolic processes [33]. The simultaneous blockade of these core catabolic routes, rather than inhibition of a single pathway, underscores BBR’s capacity to disrupt bacterial physiology at a fundamental level, making compensatory adaptation by the bacterium exceedingly difficult.

In contrast to the widespread downregulation of metabolism, a set of genes associated with flagellar assembly and DNA repair was notably upregulated. The significant upregulation of flagellar biosynthesis genes (e.g., *flgE*) may indicate an attempt to enhance motility, a behavior often linked to escape from unfavorable environments or increased surface colonization in response to threats [34]. It is noteworthy that flagellar assembly is a tightly regulated and energetically costly process for bacteria, and the upregulation of such genes under stress could represent a resource-intensive defensive strategy. However, despite this upregulation, BBR significantly suppressed biofilm formation, a key virulence trait. This suggests that the energy deficit from metabolic suppression likely impairs the construction of functional virulence structures, rendering the transcriptional upregulation ineffective at the phenotypic level and supporting the antivirulence action of BBR. Concurrently, the activation of DNA repair pathways likely represents a defensive reaction to genomic instability under BBR stress. Whether this instability is triggered by BBR-induced oxidative DNA damage or secondary effects of metabolic disturbance remains unclear and requires further experimental verification. This is supported by studies showing that berberine can indeed induce oxidative DNA damage and impair DNA repair mechanisms in other cell types [35]. Therefore, the upregulation of these defense and escape mechanisms, while indicative of severe bacterial stress, may ultimately represent a futile compensatory effort in the face of BBR’s primary action of crippling core metabolism. Notably, the prominent dysregulation of flagellar assembly and amino acid catabolic pathways observed in this study represents species-specific responses of *A. hydrophila* to berberine stress, which is closely associated with the environmental adaptation strategy of aquatic pathogenic bacteria.

## 5. Conclusions

In conclusion, this study demonstrates that berberine inhibits *A. hydrophila* through a multi-target mechanism. It primarily damages cell membrane integrity, inhibits biofilm formation and concurrently suppresses central energy metabolism pathways, inducing a cellular energy crisis. These findings provide a deeper mechanistic understanding of BBR’s antibacterial action and support its potential as a multi-functional agent for managing bacterial diseases in aquaculture. Further direct physiological validation, time-course transcriptomic analyses, and more comprehensive virulence assays are warranted in future studies to fully elucidate the underlying mechanisms.

## Figures and Tables

**Table 1:** Real-time PCR primer sequences.

Primers Name	Primer Sequences (5′→3′)	Tm Value
16S rRNA-F	AGAGTTTGATCCTGGCTCAG	55.4
16S rRNA-R	TACGGCTACCTTGTTACGACTT	55.8
*hisC-F*	TGACCTTGCTGGCATCGAAT	60.0
*hisC-R*	ACCACGATTGCTCGGTCTTT	60.1
*katE-F*	TGGTTACCGGTGATGTGAGC	60.0
*katE-R*	GGTGTAGAACTTCACCGCGA	60.0
*aerA-F*	TCTACCACCACCTCCCTGTC	59.3
*aerA-R*	GACGAAGGTGTGGTTCCAGT	59.8
*sucA-F*	ATGCTGGTGAAGGCTATCGG	59.8
*sucA-R*	TTCGATGTTGCGGTAGATGC	59.5
*ompF-F*	ATGAAAGCGTTGCTGTCGTC	59.2
*ompF-R*	GCTGCGGATAAGGTGATGGT	59.7
*ndk-F*	AGATCATCACCCGCATCGAG	59.7
*ndk-R*	ATTCGATGAGCGAGCCGAAG	60.6
*flgE-F*	TCAGCGACCTACAGCAAT	58.9
*flgE-R*	CACCAGACAGCAGAGACT	59.4
*rtxB-F*	GCCAAGAACCTGACCTAC	59.6
*rtxB-R*	TAACTACCGTCCGACCAT	59.9
*recA-F*	GGTGAAGGTCTGGTGAAGGA	59.4
*recA-R*	CGCTTCTGCTGATGGTCATA	59.1
*fliC-F*	GCAACGCTATCACCAAGACC	59.6
*fliC-R*	GTTGCGGATGTTGAGGTAGC	59.3

**Table 2:** Statistics of transcriptome sequencing data.

Sample Name	Clean Reads	Clean Error Rate (%)	Clean Q20 (%)	Genome Mapped Ratio (%)
BBR1	23,532,212	0.0118	99.27	93.39
BBR2	29,483,216	0.0118	99.25	92.97
BBR3	22,317,392	0.0119	99.24	93.48
BBR4	25,886,884	0.0118	99.27	93.67
BBR5	29,501,504	0.0118	99.25	93.95
CK1	27,596,592	0.0117	99.34	95.1
CK2	24,988,898	0.0117	99.33	95.54
CK3	24,232,532	0.0118	99.3	95.33
CK4	25,509,792	0.0117	99.35	95.01
CK5	25,403,640	0.0117	99.33	95.61

## Data Availability

The original contributions presented in the study are included in the article. Further inquiries can be directed to the corresponding author.
